# Phenylalanine Versus Tyrosine (Pos. 367/332 in MCT1/MCT4) in the Substrate Binding Site Defines Affinity and Preferred Directionality of Human Monocarboxylate Transporters 1–4

**DOI:** 10.1111/apha.70267

**Published:** 2026-06-14

**Authors:** Maike Menzel, Ioana‐Daniela Dumitru, Josh Peters, Jan‐Bernd Hövener, Andrey N. Pravdivtsev, Ana‐Nicoleta Bondar, Eric Beitz

**Affiliations:** ^1^ Department of Pharmaceutical and Medicinal Chemistry, Pharmaceutical Institute Kiel University Kiel Germany; ^2^ Faculty of Physics University of Bucharest Bucharest Romania; ^3^ Section Biomedical Imaging, Molecular Imaging North Competence Center, Department of Radiology and Neuroradiology University Hospital Schleswig‐Holstein, Kiel University Kiel Germany; ^4^ Forschungszentrum Jülich Institute of Computational Biomedicine (INM‐9) Jülich Germany; ^5^ Faculty for Interdisciplinary Sciences University of Bucharest Bucharest Romania; ^6^ Institute for Research of the University of Bucharest, ICUB Bucharest Romania

**Keywords:** affinity, binding site, directionality, hyperpolarization, inhibitor, lactate, mutation, transport

## Abstract

**Aim:**

Human monocarboxylate transporters 1–4, MCT, are key for the lactate/H^+^ exchange between glycolytic and oxidative cancer cells, white and red muscle fibers, or in the astrocyte‐neuron shuttle. The common MCT transport mechanism involves three conserved residues, that is, a substrate‐attracting Lys and a conformation‐locking Asp/Arg salt bridge (positions 38 and 309/313 in MCT1). Yet, it remained unclear which sites define isoform‐specific substrate affinity and preferred transport directionality.

**Methods:**

Here, we analyzed structural differences in the binding sites of MCT1–4 and determined their impact on the biophysical transport properties and inhibitor binding using radiolabeled transport assays in yeast.

**Results:**

We found differences in amino acid positions with sidechain hydroxyl groups. While the higher affinity MCT1 and MCT2 carry Phe/Ser‐OH (pos. 367/371 in MCT1), the lower affinity MCT3 and MCT4 have Tyr‐OH/Gly. Mutation of Phe/Ser‐OH in MCT1 to Tyr‐OH/Gly markedly decreased the affinity for lactate and pyruvate, while it did not change the affinity for propionate. The maximal transport velocity increased with decreasing affinity, and the preferred transport directionality shifted toward export. Likewise, replacing Met151 by Ala shifted transport bias possibly by eliminating conformation‐stabilizing sulfur‐aromatic interactions. Moreover, these mutations lowered the activity of the clinical candidate MCT1 inhibitor AZD3965 by three orders of magnitude providing insight into the molecular drug binding mode and explaining the strong preference for MCT1 over MCT4.

**Conclusions:**

Together, subtle changes in the arrangement of sidechains in the MCT binding site determine basic monocarboxylate/H^+^ transport properties that impact lactate‐related physiology, namely cellular metabolism, reprogramming, and signaling.

## 1 Introduction

Lactate, long thought to be solely a waste product derived under hypoxic metabolic conditions, has gained attention as a key metabolite and signaling molecule in health and disease [1, 2, 3]. A century ago, cellular secretion of lactate despite sufficiently supplied oxygen was found to be a trait of tumorous tissues [4], now termed the Warburg effect. Warburg cells derive energy from the less efficient glycolysis pathway rather than from oxidative phosphorylation. Ample amounts of lactate generated during the process must be secreted in order to maintain glycolysis and prevent toxic cellular acidification [5]. Later, an interplay with neighboring tumor cells undergoing oxidative phosphorylation was described [6, 7]. Such cells profit from the uptake of lactate, followed by oxidation to pyruvate, which can then be metabolized in the citric acid cycle. Today, lactate shuttling between producing glycolytic cells and consuming oxidative cells is seen as a fundamental principle in the physiology of for example, muscle (from white to red muscle fibers) and brain (astrocyte‐neuron shuttle) [8, 9].

**FIGURE 1:**
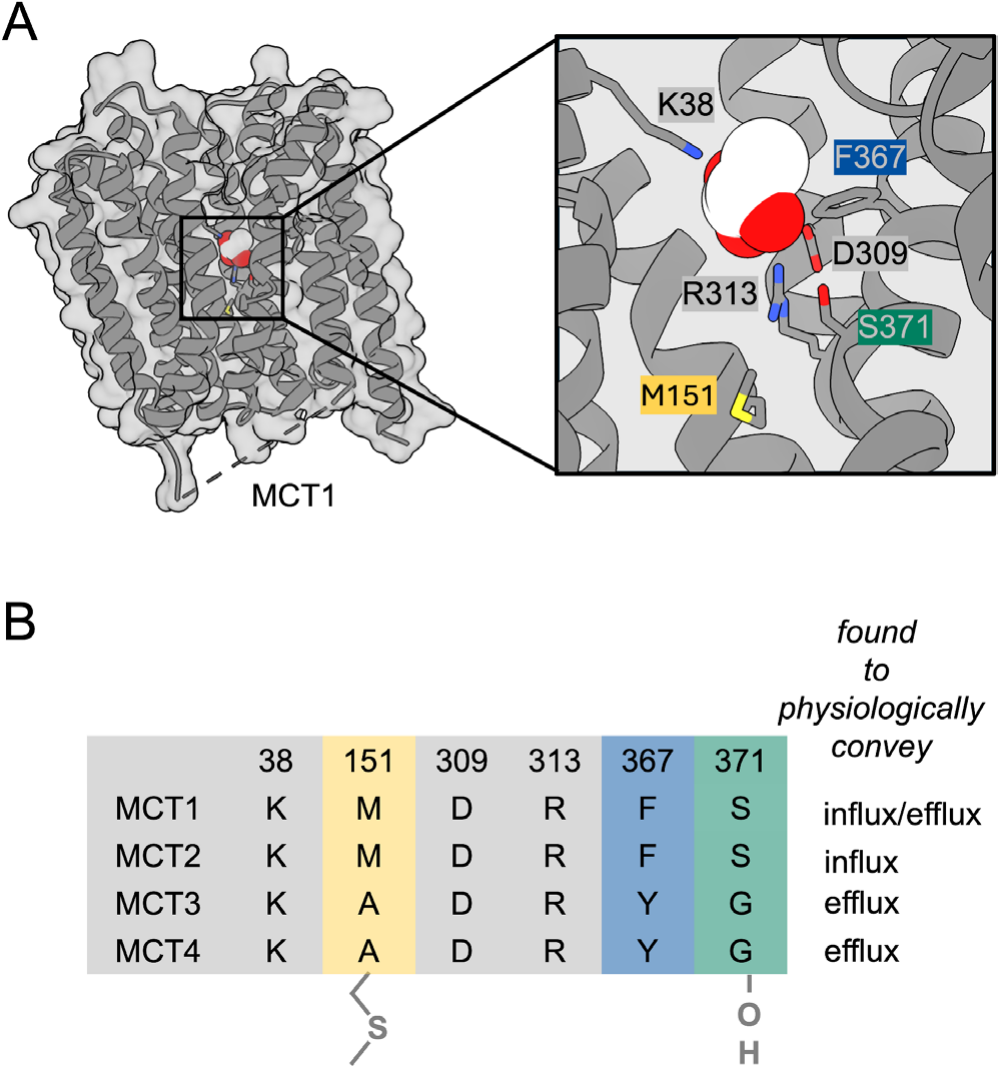
Structural differences in lactate transporting MCT1‐4. (A) The MCT1 protein structure (PDB# 6LZ0) is depicted with a focus on residues of the lactate binding site. Deviating sites between MCT1/2 and MCT3/4 are colored. (B) Amino acid composition of the lactate binding sites of MCT1‐4 with MCT1 numbering.

The directionality of lactate use and production is governed by the associated cellular metabolism. The resulting transmembrane gradients and pH conditions determine the kinetics of involved transport proteins. Mainly four monocarboxylate transporters, MCT1–4, from the solute carrier family SLC16A facilitate proton‐coupled transmembrane lactate transport in humans [10]. Besides the prevailing gradients, protein properties such as substrate affinity and transfer mechanics of the MCT isoform determine the velocity and preferred directionality of transport. MCTs operate by an alternating access mechanism that switches between outward‐open and inward‐open conformations upon substrate/H^+^ binding [11]. A conserved motif of three charged amino acids, Lys38, Asp309, and Arg313 (MCT1 numbering; Figure 1A), was found to be essential for transport functionality [11, 12]. According to the current view [13], the positively charged Lys38 attracts the lactate anion. After binding, lactate itself transfers a proton from Lys38 to the Asp309/Arg313 salt bridge. Breakage of the salt bridge initiates the conformational change, releasing the lactate and the proton in a 1:1 stoichiometry on the *trans* side.

Although MCT1–4 most presumably share this transport mechanism [12], in a physiological setting, the MCT isoforms exhibit differences regarding their preference for lactate import or export (Figure 1B). This depends in part on the presence and state of transport‐supporting proteins such as basigin [14, 15], a transmembrane protease [16, 17], and carbonic anhydrases [18, 19]. According the principles of thermodynamics, a secondary active transport system can maximally reach even concentrations of substrate on either side of the membrane [20]. The inward and outward transport velocities of a transporter can differ, which we term here preferred directionality. An MCT protein‐internal property affecting the preferred transport directionality is the isoform‐specific lactate binding affinity (Figure 1B) [21]. The almost ubiquitously expressed MCT1 exhibits lactate affinity in the range of the physiological lactate concentration (*K*_m_ 3–5 mM), and mediates both, import and export. High‐affinity MCT2 is predominantly found in tissues that depend on the uptake of lactate as a gluconeogenic precursor (e.g., liver parenchymal cells) or to fuel mitochondrial respiration (e.g., neurons). The apparent *K*_m_ of MCT2 for lactate of 0.7 mM is below the typical extracellular lactate concentration ensuring a constant import rate [22]. MCT3 and MCT4, in turn, were found in tissues relying on lactate export. Low lactate affinity would enable export into high‐lactate environments [23]. However, earlier measurements of MCT4 in *Xenopus laevis* oocytes yielding a *K*_m_ of 28–35 mM [23, 24] were recently challenged. Using heterologous MCT4 expression in human HEK293, MDA‐MB‐231 cells, and macrophages revealed high lactate affinity (0.7–1.7 mM) [25]. The authors conducted mathematical modeling and claimed that even high lactate affinity would allow for lactate export into extracellular environments containing high lactate concentrations.

Variability in the observed transport properties of MCT1–4 is possibly due to the investigated cellular context, which prompted us to analyze the intrinsic structural properties of the MCT binding site and their role in defining transport kinetics. To avoid interference from metabolism and MCT‐modulating proteins, we used *Saccharomyces cerevisiae* yeast as a microbial, non‐lactate metabolizing model organism. Using radioactive ^14^C labels and ^13^C nuclear magnetic resonance of hyperpolarized tracers allowed us to investigate the transport mechanism for 30 min with a resolution of 5–10 min, and for 300 s with a resolution of seconds. The yeast system enabled largely background‐free determination of MCT transport kinetics from the seconds timeframe (initial rate, *K*_m_, *v*_max_) into the establishment of the transmembrane lactate/H^+^ equilibrium (*k*_in_/*k*_out_ ratio). We analyzed MCT1 variants with point mutations in the lactate binding site mimicking structural features of MCT4. A key finding was the exchange of Phe367 for Tyr in MCT1, that is, effectively adding a single hydroxyl group, alters the intrinsic transport properties to those of MCT4 in terms of substrate affinity, preferred transport directionality, and inhibitor binding.

## 2 Results

### 2.1 The MCT1 and MCT4 Substrate Binding Sites Exhibit Different Arrangements of Hydroxyl Groups

Initially, we compared the amino acid sequences of MCT1–4 in the context of the resolved human MCT1 and MCT2 protein structures [26, 27] to identify distinct amino acid residues in the substrate binding sites (Figures 1B and S1). This revealed three positions that distinguish the higher affinity MCT1 and MCT2 (Met151, Phe367, Ser371; MCT1 numbering) from the putatively lower affinity MCT3 and MCT4 (Ala, Tyr, Gly) (Figure 1B). Of these amino acid positions, Met151 may have a more structural role by defining the end of the binding site toward the cytoplasmic side. It had been previously implicated in contributing to the binding of the MCT1 inhibitor AZD3965 [26]. Phe367 and Ser371 are close to the bound monocarboxylate substrate and presumably make direct contact. An MCT1 variant carrying an F367Y mutation was generated before [26, 28]. It reportedly lost the ability to transport pyruvate [26] and showed lower activity in lactate and oxoproline transport [28]. However, more in‐depth analyses are missing.

### 2.2 Yeast Cells Enable Equilibrium Studies in Transmembrane Lactate Transport

**FIGURE 2:**
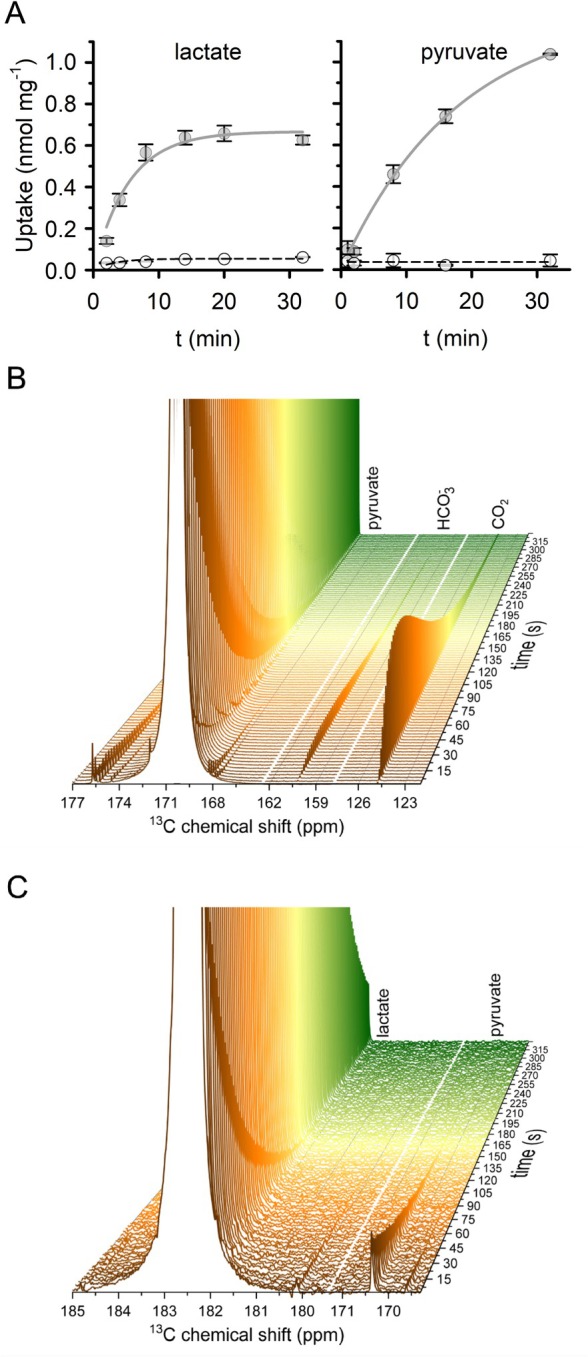
MCT1‐facilitated uptake and metabolism of lactate and pyruvate in *S. cerevisiae* . (A) Uptake curves of ^14^C‐spiked pyruvate and lactate from 1 mM inward gradients indicate establishment of a transmembrane equilibrium for lactate transport after 15 min but not for pyruvate. Background transmembrane diffusion was minimal (dashed lines). (B) High resolution ^13^C NMR spectroscopy of hyperpolarized [1–^13^C]‐pyruvate (170.5 ppm) showed rapid metabolic conversion to H^13^CO_3_^−^ (160 ppm) and ^13^CO_2_ (125 ppm) with a mean rate constant of 3.02 ± 0.03 × 10^−4^ s^−1^. Generally, the ratio of CO_2_/HCO_3_^−^ depends on pH (here 5.7). No pyruvate to lactate conversion was observed. (C) High resolution ^13^C NMR spectroscopy of hyperpolarized [1–^13^C]lactate (182.5 ppm) and its metabolic product [1–^13^C]pyruvate (170.5 ppm). The dynamics of the pyruvate signal were well‐fitted by a tri‐exponential decay function, indicating a net influx from lactate to the pyruvate pool with a rate constant of 1.39 ± 0.16 × 10^−5^ s^−1^. The amount of lactate converted to pyruvate was (59 ± 8)‐times lower compared to the conversion of pyruvate to CO_2_/HCO_3_^−^ within the same timeframe. See Supporting Information for details and the extracted kinetics.

We expressed human MCT1 in *S. cerevisiae* (W303‐1A jen1Δ ady2Δ) lacking endogenous monocarboxylate transporters (Figure S2) [14, 29]. Uptake of ^14^C‐labeled lactate and pyruvate from 1 mM inward gradients was measured over a time span of 32 min (Figure 2A). Background uptake by transmembrane diffusion was minimal in the used yeast strain and subtracted from uptake via MCT1. From the initial, rapid phase we determined transport rates of 0.12 ± 0.04 nmol mg^−1^ min^−1^ for lactate and 0.07 ± 0.02 nmol mg^−1^ min^−1^ for pyruvate. After about 15 min, lactate transport reached a transmembrane equilibrium at 0.67 ± 0.03 nmol lactate mg^−1^ yeast (Figure 2A). The apparent intracellular concentration of pyruvate continued to increase without arriving at a plateau (Figure 2A).

We assumed that lactate would remain metabolically unaltered over the assay time, allowing for a transport equilibrium to establish, whereas pyruvate would be rapidly consumed by entering the energy metabolism. To test this directly, we followed up on the metabolic fate of pyruvate and lactate in yeast using hyperpolarized ^13^C NMR spectroscopy (Figure 2B,C). Hyperpolarization is a technique to magnetically label selected nuclei in various molecules so that the labeled nucleus can then be followed across metabolism with high temporal resolution, but only for a limited time [30, 31]. Importantly, the resonance frequency of the nucleus (given in relative units, ppm) allows identifying the emitting molecule. When adding 56.4 mM hyperpolarized ^13^C‐pyruvate to the yeast, we detected rapid metabolic conversion to hyperpolarized ^13^CO_2_ and H^13^CO_3_^−^ (Figure 2B). The pyruvate consumption rate per 1 billion cells was 3.02 ± 0.03 × 10^−4^ s^−1^ (Figure S3A), being affected by a combination of cellular uptake and metabolism. The addition of 20.8 mM hyperpolarized ^13^C‐lactate (and some copolarized pyruvate) in turn showed only little metabolism as no other hyperpolarized ^13^C‐metabolites were identified (Figure 2C). Analyzing the pyruvate signal yielded a tri‐exponential decay, which indicated an influx from hyperpolarized lactate to hyperpolarized pyruvate with a conversion rate per 1 billion cells of 1.39 ± 0.16 × 10^−5^ s^−1^ (Figure S3B). Therefore, the lactate conversion was 59 ± 8‐times lower compared to pyruvate.

Extrapolating these results to a typical ^14^C experiment, about 2.78% ± 0.03% and 0.13% ± 0.01% of 1 mM pyruvate and 1 mM lactate, respectively, would be consumed by 48 million cells in 32 min. Note that the reaction temperature in the ^13^C experiment was 37°C, which may have led to faster transport and metabolism compared to the 19°C used in ^14^C experiments.

### 2.3 Mutation of MCT1 Phe367 to MCT4‐Like Tyr Shifts Transport Equilibrium Toward Export

**FIGURE 3:**
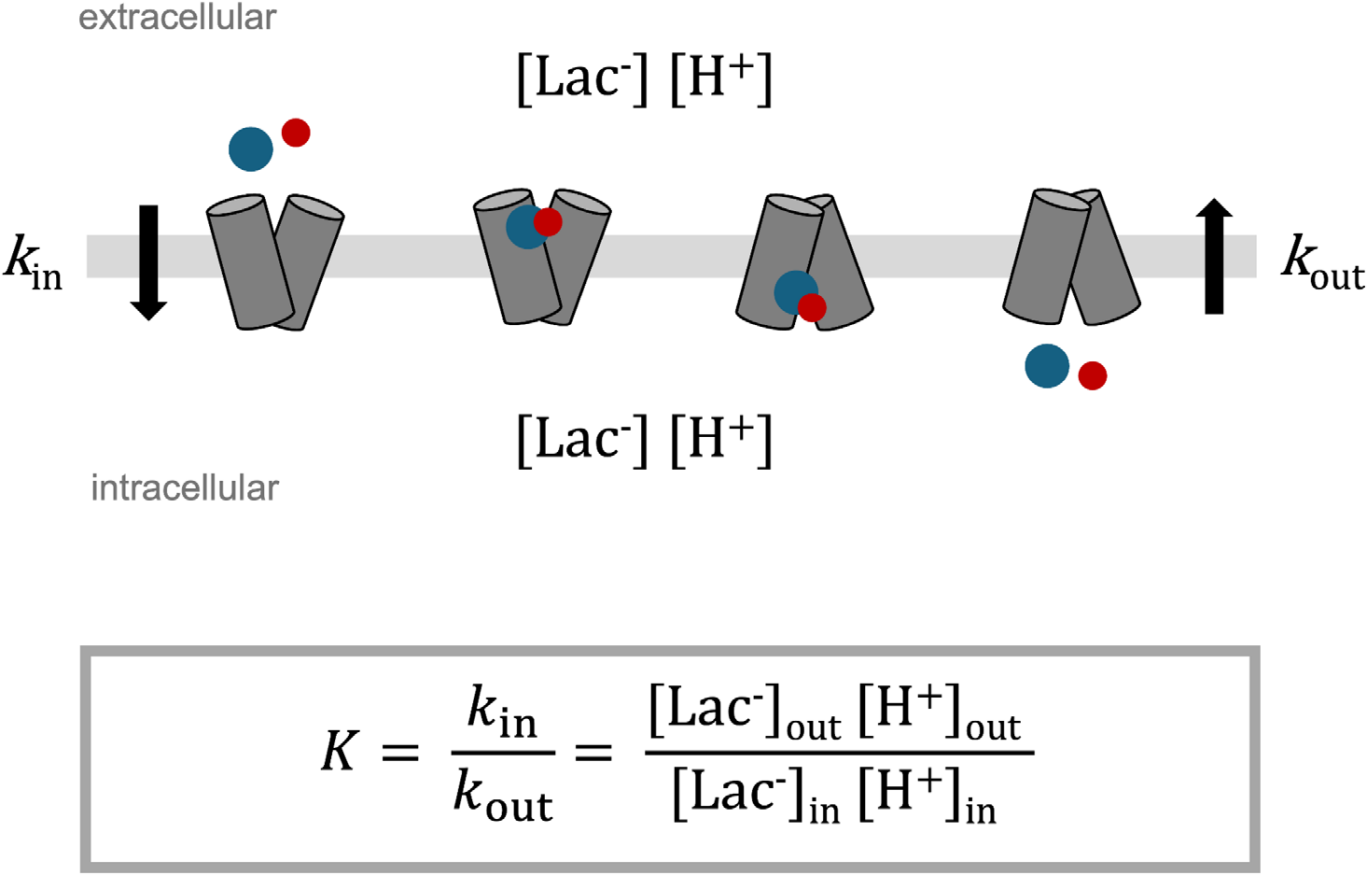
The transmembrane lactate equilibrium position depends on the pH gradient and protein intrinsic kinetic transport properties, *k*_in_ and *k*_out_.

The position of the transport equilibrium, *K*_eq_ equals the balanced ratio of the kinetic constants of inward and outward transport: *K*_eq_ = *k*_in_/*k*_out_ allowing one to conclude on the preferred transport directionality of the studied transporter (Figure 3). In the case of lactate/H^+^ transport via an MCT, the kinetic constants depend on the substrate and proton concentrations in the intra‐ and extracellular space, and on protein‐intrinsic properties, mainly substrate affinity (*K*_m_) and the maximal transport velocity (*v*_max_). The equilibrium position, however, is independent of the number of transporters present at the plasma membrane. Therefore, direct comparisons of the transport capacities elicited by different MCT mutation constructs are valid even if their expression levels vary.

**FIGURE 4:**
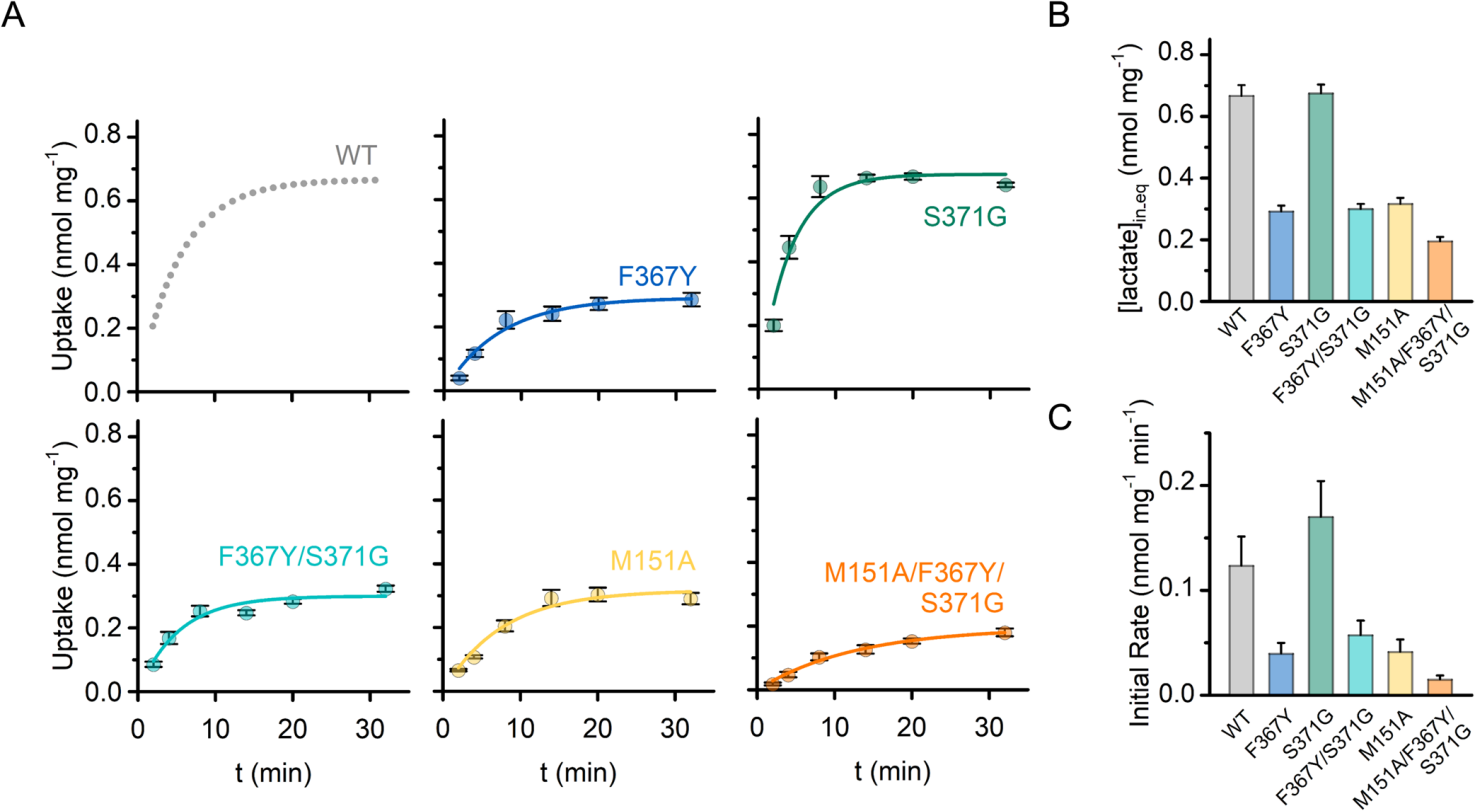
Lactate uptake into yeast via MCT1 wildtype (WT) and MCT1 mutants. (A) Uptake from 1 mM inward gradients at pH 6.8 were monitored for 32 min, and curves were fitted to an exponential‐rise‐to‐maximum function (*r* ^2^ ≥ 0.94). Error bars denote SEM. (B) The intracellular lactate concentrations at transport equilibrium. (C) The bars depict initial lactate transport rates. Error bars denote the standard error of the fit.

We generated five MCT1 mutants carrying either single (M151A, F367Y, S371G), double (F367Y/S371G), or triple mutations (M151A/F367Y/S371G) to mimic an MCT4‐like substrate binding site. All constructs were expressed in yeast (Figure S2). For the transport assays (Figure 4A), an inward gradient of 1 mM lactate was used, and the external buffer pH was adjusted to the intracellular yeast pH of 6.8 to omit a transmembrane pH gradient. Of the five MCT1 mutants, only S371G displayed unaltered properties compared to wildtype MCT1 regarding the transport equilibrium position (Figure 4B) and initial transport rate (Figure 4C). The MCT1 F367Y mutation that adds a single hydroxyl group to the transporter protein decreased the intracellular lactate equilibrium concentration by 56% (Figure 4B) and the initial velocity by 70% (Figure 4C). With respect to the underlying *k*_in_/*k*_out_‐ratio this means that the preferred transport directionality has about doubled toward export relative to the import direction. The joint mutation of F367Y with S371G showed the same decrease in intracellular lactate as F367Y alone (Figure 4B), indicating that Phe367 is mainly responsible for the shift in the preferred transport directionality. MCT1 M151A showed a similar decrease (53%) as the F367Y mutation, and the combination as present in the triple mutant (M151A/F367Y/S371G) was additive, yielding an 80% lower intracellular lactate concentration (Figure 4B).

**FIGURE 5:**
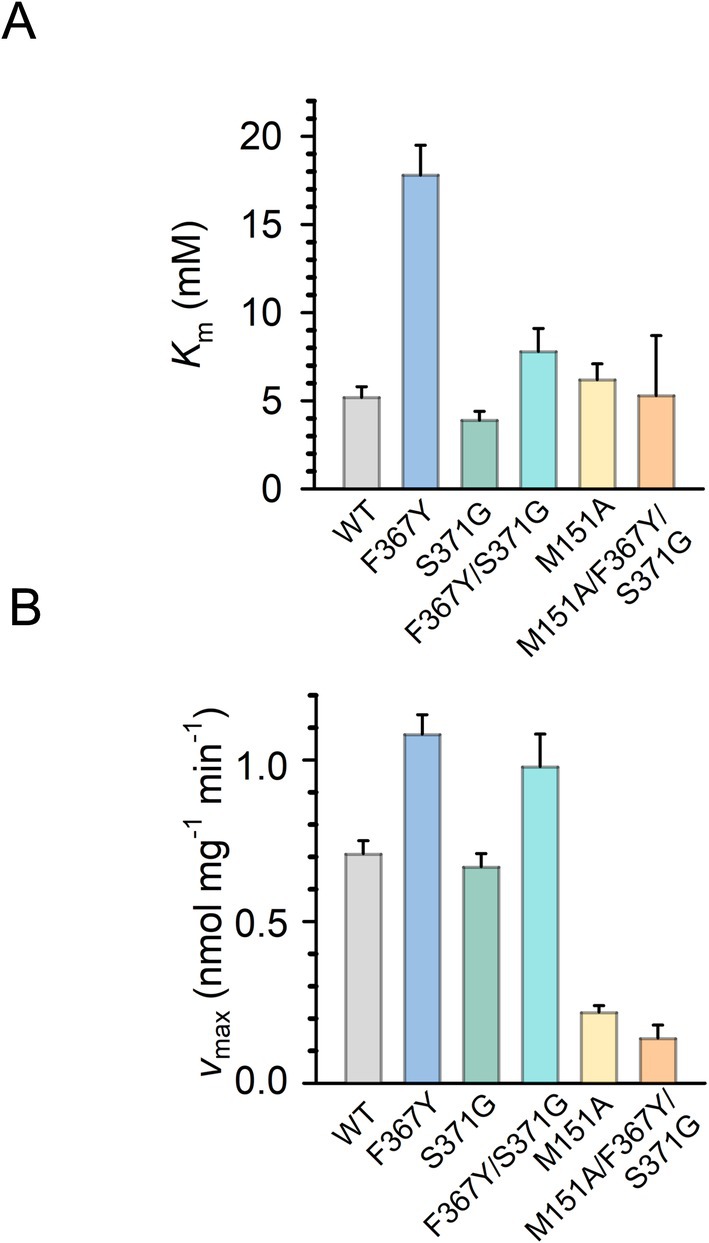
Michaelis–Menten kinetics of MCT1 wildtype (WT) and mutants. (A) Shown are the apparent lactate affinities expressed as *K*_m_. Higher values indicate lower affinity. (B) The bars depict the maximal transport velocity, *v*_max_, Error bars denote SEM.

To relate the observed shift in the *k*_in_/*k*_out_‐ratio to the Michaelis–Menten kinetics of the MCT1 variants, we determined *K*_m_ and *v*_max_ for each mutant (Figures 5 and S4). In line with an unchanged transport equilibrium position, MCT1 S371G showed no significant change in *K*_m_ (5.3 mM) and *v*_max_ (0.71 mM) values, which were comparable to those of the wildtype. However, MCT1 F367Y decreased the lactate affinity more than 3 times (*K*_m_ to 17.8 mM). Concurrently, *v*_max_ increased 1.5 times to 1.08 nmol mg^−1^ min^−1^. In the MCT1 F367Y/S371G double mutant, affinity was partly recovered (*K*_m_ 7.8 mM), while *v*_max_ remained high at 0.98 nmol mg^−1^ min^−1^. In accordance with a structural role rather than direct substrate binding, the MCT1 M151A mutation did not significantly affect lactate affinity (*K*_m_ 6.1 mM) but strongly decreased *v*_max_ (0.21 nmol mg^−1^ min^−1^) possibly by interfering with the conformational flexibility of the protein during the transport cycle [26], and to its location close to the transporter hinge region [32].

**FIGURE 6:**
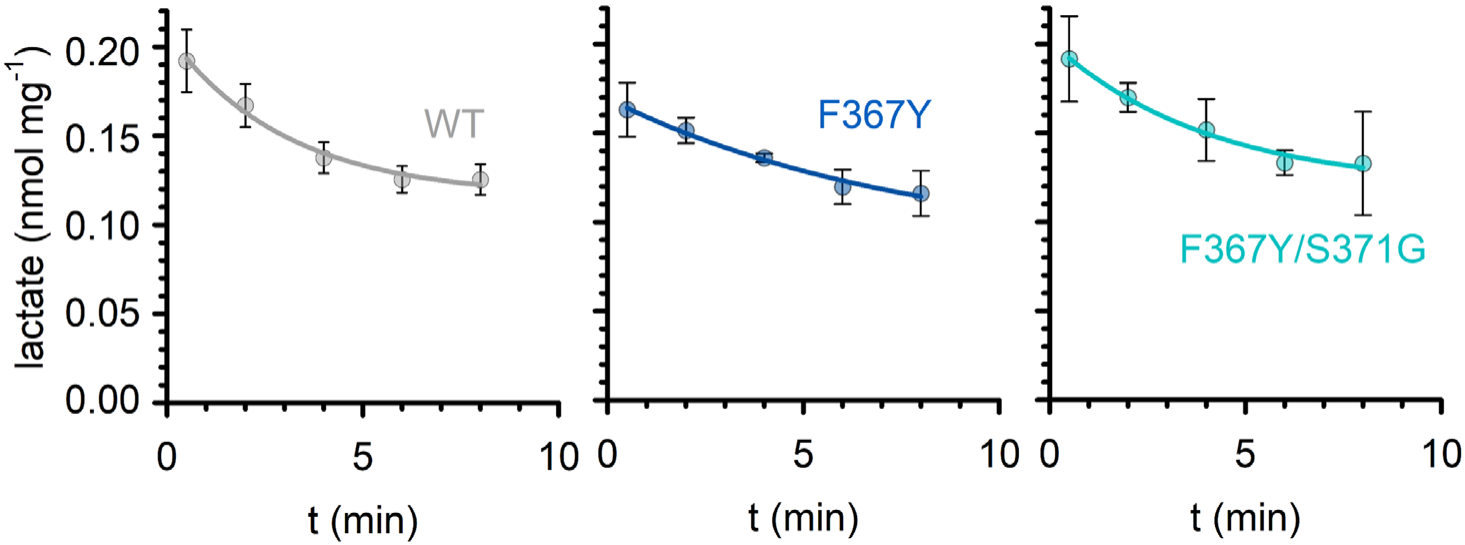
Lactate transport in the export direction via yeast‐expressed MCT1 wildtype (WT) and mutants. Cells were preloaded with ^14^C‐labeled lactate and transferred into lactate free buffer. The curves were fitted to an exponential‐decay function (*r* ^2^ ≥ 0.98). Error bars denote SEM. of technical duplicates.

To complement the lactate uptake data, we assayed lactate transport via MCT1 wildtype, MCT1 F367Y, and MCT1 F367Y/S371G in the export direction (Figure 6). Therefore, we preloaded yeast with ^14^C‐lactate to about 0.2 nmol mg^−1^ and subsequently placed the cells in lactate‐free buffer. Contrary to the import direction, which showed slower transport of the MCT1 F367Y mutants, the export rates were not significantly different from MCT1 wildtype. Relating the export data to the import rates therefore confirms the shifted transport equilibrium toward export in the MCT1 F367Y mutants.

### 2.4 Collision of the Added F367Y Hydroxyl With a Substrate Oxygen May Explain Decreased Affinity

**FIGURE 7:**
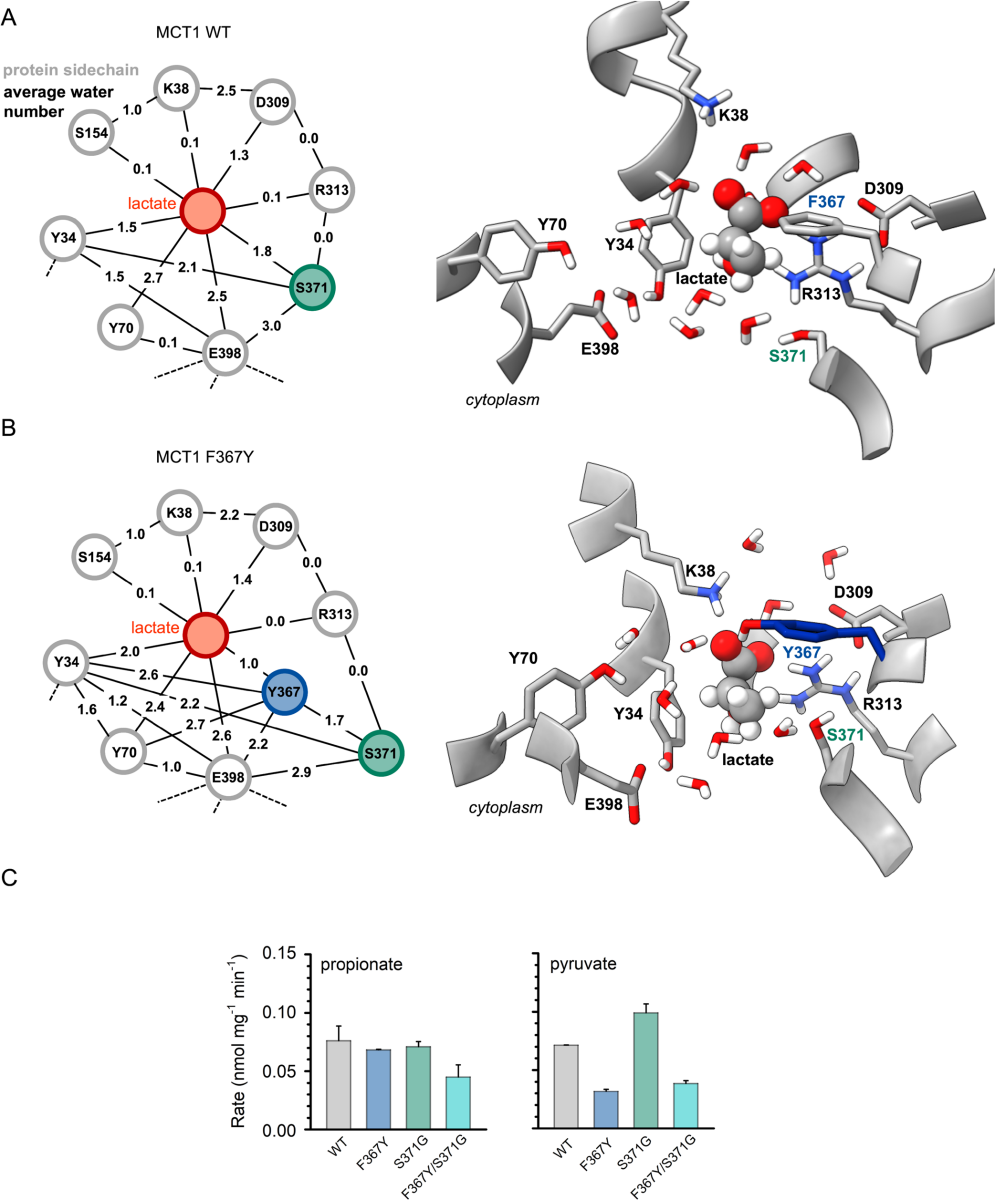
Hydrogen bond cluster of the lactate bound to MCT1, and transport of propionate and pyruvate. (A, B) Graph representations of the lactate hydrogen bond clusters (left) and molecular graphics of the lactate binding site in MCT1 wildtype (WT) and MCT1 F367Y. The numbers along the graph edges indicate the average number of water molecules in a bridge. Note that average water numbers < 1 indicate that the hydrogen bond is mostly direct between the two residues (graph nodes), whereas water numbers > 1 indicate a mostly water bridged connection. The graph nodes for Tyr367 and Ser371 are shown as circles filled blue and green, respectively. The molecular graphics on the right side are based on coordinate sets from the corresponding MD simulations. Note that the E398 has additional hydrogen bond connections, which are shown in Figures S6 and S7. (C) The bars depict initial transport rates for propionate and pyruvate transport via yeast‐expressed MCT1 and mutants.

To gain insight into the molecular mechanism that determines substrate affinity in the MCT1 wildtype and F367Y mutant, we carried out atomic‐level simulations of wildtype and mutant MCT1 in hydrated lipid bilayers; each simulation was performed in duplicate and extended to 0.5 μs. We used the simulation trajectories to compute the hydrogen bond cluster of the lactate using the graph‐based algorithm Bridge/Bridge2 [33, 34]. In Figure 7A,B, left we illustrate the cluster of hydrogen bonds and water‐mediated bridges that are sampled during at least 50% of the time; numbers along the edges give the average length of the water‐mediated bridges between sidechains (replicas in Figures S5 and S6). In MCT1 wildtype, the lactate molecule sampled three direct hydrogen bonds (distance < 1 water) with the sidechains of Lys38, Ser154, and Arg313, and five water‐mediated bridges (distance > 1) with Tyr34, Tyr70, Asp309, Ser371, and Glu398 (Figure 7A, left). In the MCT1 F367Y mutant, we found Tyr367 to largely occupy the former space of Ser371 and water‐bridging to lactate; it further recruits additional bridges to Tyr34, Tyr70, Ser371, and Glu398 (Figure 7B, left). The molecular structure representation of the simulation shows a slightly rotated orientation of lactate in the binding sites of MCT1 wildtype and the MCT1 F367Y mutant, which we hypothesize might affect affinity (Figure 7A,B, right).

To address this notion experimentally, we measured the transport rates for propionate and pyruvate with MCT1 wildtype and the MCT1 F367Y, S371G and F367Y/S371G double mutant. Propionate lacks an oxygen at the α‐carbon and should be unaffected by the F367Y mutation, whereas the α‐keto group of pyruvate should behave similarly to lactate. Indeed, the transport rate of pyruvate was decreased by 56% (MCT1 F367Y) and 47% (MCT1 F367Y/S371G), whereas propionate transport remained unaffected within the error margins (Figure 7C). The MCT1 S371G mutation did not impede propionate or pyruvate transport (Figure 7C). This corroborates the proposed collision of the tyrosine hydroxyl with lactate and pyruvate, and likely with other MCT more bulky substrates including β‐hydroxybutyrate, acetoacetate and even oxoproline carrying the oxygen in a γ‐lactam structure.

### 2.5 Activity of the MCT1 Inhibitor AZD3965 Decreases With M151A, F367Y, and S371G Mutations in an Additive Manner

AZD3965 is a potent, nanomolar inhibitor of MCT1 exhibiting cross‐activity on MCT2, yet it has a minimal effect on MCT4 [35, 36]. The binding mode to MCT1 was determined by cryo‐electron microscopy revealing four interacting residues that differ from MCT4, namely Met151, Leu281, Phe367, and Ser371 [26]. Functional analyses showed that mutation of Leu281 abolished the activity of AZD3965, whereas replacement of Met151 or Ser371 slightly decreased activity by a factor of 2. The MCT1 F367Y mutant could not be tested by the authors due to too low transport functionality. Since in our hands transport of the MCT1 F367Y mutant was sufficiently high, and this site turned out to be decisive to render transport properties of MCT1 toward MCT4‐like, we assayed the effect on AZD3965 activity. We further tested the MCT1 M151A and S371G single mutants and combinations for inhibition by AZD3965.

**FIGURE 8:**
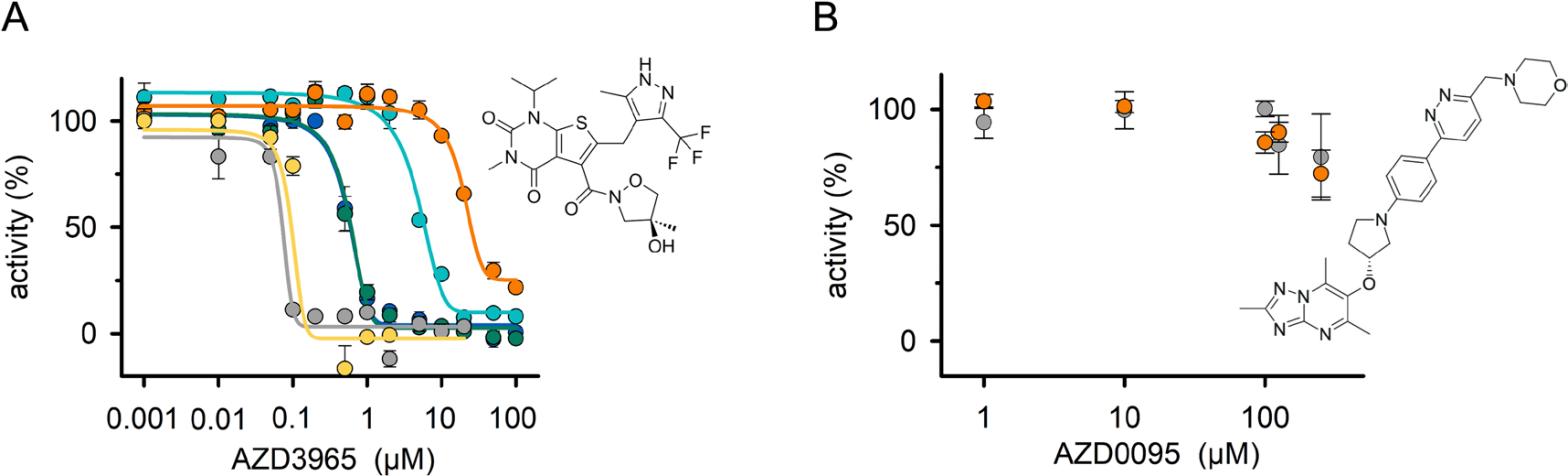
Action of MCT1 and MCT4 inhibitors on yeast‐expressed MCT1 and mutants. (A) IC_50_ determinations (*r* ^2^ ≥ 0.97) of the MCT1 inhibitor AZD3965 show decreased binding in MCT1 binding site mutants: MCT1 wildtype (gray), M151A (yellow), F367Y (blue), S371G (green), F367Y/S371G (turquoise), M151A/F367Y/S371G (orange). (B) The low activity of the MCT4 inhibitor AZD0095 remained unaffected by the triple mutation of MCT1 wildtype (gray) to M151A/F367Y/S371G (orange). Error bars denote SEM.

In line with the previous study, the M151A exchange hardly affected AZD3965 activity (Figure 8A, yellow curve). However, the MCT1 F367Y and S371G single mutants equally decreased AZD3965 activity by one order of magnitude, and the combination was additive leading to a shift by two orders of magnitude (Figure 8A, blue, green and turquoise curves, resp.). The MCT1 M151A/F367Y/S371G triple mutant decreased AZD3965 activity even further (Figure 8A, orange curve). The data show that the discriminating Phe367/Ser371 positions between MCT1 and MCT4 are major AZD3965 interaction sites that similarly and jointly contribute to inhibitor activity.

Recently, a potent nanomolar MCT4 inhibitor, AZD0095, was discovered [37]. Selectivity over MCT1 is reportedly greater than 1000 times. It is structurally unrelated to the MCT1 inhibitor AZD3965, and its binding site in MCT4 is unknown. We tested the MCT1 M151A/F367Y/S371G triple mutant for inhibition by AZD0095 to test for interactions at the substrate binding site (Figure 8B). MCT1 wildtype and the triple mutant, however, showed equally low inhibition by AZD0095. This indicates that AZD0095 is likely an allosteric rather than a competitive inhibitor of MCT4 lactate transport.

## 3 Discussion

Our findings indicate that hydrogen bond interactions between the substrate and hydroxyl groups in the MCT binding site determine key properties of transport, including affinity, and inward/outward velocities. There are only a few examples that experimentally demonstrate a biased transport directionality of secondary active transporters. One is transport of glutamate via the excitatory amino acid carrier 1, EAAC1 [38]. Here, transport of glutamate with a net charge of −1 is co‐transported with three Na^+^ ions. Sodium co‐transport strongly drives transport in the inward direction due to the steep inward transmembrane Na^+^ gradient. This overcompensates for the repulsion of the negative charge of glutamate by the negative resting membrane potential. Nevertheless, EAAC1 glutamate transport also occurs in the outward direction, that is, against the electrochemical gradient of Na^+^. The authors provide evidence that under transmembrane steady‐state conditions glutamate export is faster and less voltage‐dependent than import. They suggest a model in which the order of binding events with respect to Na^+^ and glutamate is responsible for the observed asymmetry. Another example also involves amino acid transport, albeit lysine with a net positive charge is transported by the yeast proton‐coupled lysine permease, Lyp1, which exhibits a strong preference for import [39]. Transport is driven by a proton inward gradient in combination with the favorable negative membrane resting potential. However, upon dissipation of the inward‐directed proton motive force, Lyp1 lysine transport in the export direction remains minimal. The authors generated equilibrium conditions in Lyp1‐containing proteoliposomes and determined highly asymmetric substrate affinity for the inward (μM range) and outward transport directions (mM range). They later identified sequence stretches in the extracellular loops of Lyp1 that modulate lysine affinity in an asymmetric fashion [40].

In the case of MCTs, a net neutral substrate for example, lactate/H^+^ in a 1:1 stoichiometry is transported. Accordingly, the membrane potential is irrelevant for the transport process. We further adjusted the buffer pH to match the intracellular pH in our assay setup in order to avoid a directing transmembrane proton motive force. By keeping the amino acid positions (Lys38, Asp309, Arg313) the principal transport mechanism most likely remained unaltered. Still, the lactate transport appeared asymmetric, and shifted after the introduction of certain point mutations in the binding site of MCT1. Thermodynamics dictate that the intracellular concentration in our experiments cannot exceed the extracellularly provided 1 mM of lactate. It should be noted that unless all elements of the Haldane relationship have been measured, that is, lactate concentration inside and out and proton concentration inside and out, accumulation of lactate can be apparent. In the given 1 mM range, the shift in the equilibrium position in the steady‐state compared to MCT1 wildtype was about 2 times with MCT1 F367Y, and 5 times with the MCT1 M151A/F367Y/S371G triple mutant. One can calculate the change in the Gibbs energy, ΔΔ*G*, associated with the shifted equilibrium positions of wildtype (*K*_wt_) and mutant MCT1 (*K*_mut_) using ΔΔ*G* = *R T* ln(*K*_wt_/*K*_mut_) with *R* being the gas constant (8.314 J mol^−1^ K^−1^) and *T* the absolute temperature (291 K in the assays). This results in ΔΔ*G* values of 1.68 kJ mol^−1^ (MCT1 F367Y) and 3.89 kJ mol^−1^ (MCT1 M151A/F367Y/S371G). These energy portions are smaller than the binding energy contained in a single hydrogen bond (> 8 kJ mol^−1^). It is therefore conceivable that differences in the arrangement and binding angles in the hydrogen bond network around the lactate substrate in the MCT isoforms can account for the observed differences in the transport kinetics. Asymmetry may derive from conformational differences at the binding site in the outward and inward open states of the transporter.

In addition to putatively asymmetric substrate binding affinities, different outward and inward open probabilities due to stabilization of one conformation could also generate a preference in transport directionality. In this regard, the MCT1 M151A mutant may eliminate a sulfur‐aromatic interaction which was found to contribute an energetic benefit of up to 6.3 kJ mol^−1^ [41]. Two aromatic rings of Tyr34 and Phe375 are located within 5–6 Å to the Met151 sulfur atom (Figure S7). Such an interaction could contribute to the stabilization of the outward open conformation in MCT1 and MCT2 favoring import, as opposed to MCT3 and MCT4, which lack a sulfur‐containing residue in this region.

Using high‐resolution ^13^C NMR allowed us, for the first time, to access the metabolism with much higher temporal resolution than before. The upper limit of the temporal resolution is the repetition time of the experiments, usually of the order of 100 ms (mostly for data collection). On the other side, the lower limit is given by the decay of the hyperpolarized state. This decay is likely the strongest impediment of the method, as it overlays all metabolic conversions. We accounted for this by fitting both the exchange and decay rates. Thus, using methods providing a time resolution of minutes (^14^C) or seconds (hyperpolarized ^13^C) provided unique insights complementing uptake studies (^14^C) with subsequent metabolic conversion. Our data confirm that yeast as an alcohol rather than a lactic acid fermentation organism provides the opportunity to study lactate transport in the equilibrium state, which would not be achievable in lactate metabolizing systems including mammalian cells.

In previous studies on MCT1, we obtained evidence that the bound substrate itself accepts and relays the co‐transported proton [13]. Transport is thus coupled to chemical protonation/dissociation reactions, which may also induce asymmetry depending on the inward/outward state of the transporter. Finally, we and others found that the presence of the extracellular immunoglobulin‐like domain, Ig‐I, of the MCT‐associated protein basigin affects the equilibrium position of transport via MCT1 [14] and MCT4 [16]. Basigin with an intact Ig‐I domain promotes lactate import, whereas removal of the Ig‐I domain by a transmembrane protease [16] or artificial truncation shifts transport toward export by a factor of 4–5 [14]. The mechanism by which the extracellular domain of basigin generates asymmetry in MCT transport is not fully understood. It may facilitate substrate binding to the outward‐open MCT conformation or promote proton shuttling in the extracellular vestibule of the MCT by a dielectric effect similar to microbial monocarboxylate transporters of the formate/nitrate transporter family, FNT [42].

Most likely an interplay of the above‐mentioned mechanisms conveys asymmetry in MCT transport. Asymmetric transport properties of MCT1/MCT2 favoring import and MCT3/MCT4 favoring export are in line with their physiological tasks in lactate/H^+^ shuttling [10]. While MCT1 and MCT2 primarily enable the uptake of lactate to fuel the citric acid cycle and oxidative phosphorylation, MCT3 and MCT4 serve as lactate exporters in the retinal pigment epithelium (MCT3) and in highly glycolytic tissues such as white muscle fibers and cells underlying the Warburg effect (MCT4) [12]. In the tumor microenvironment, lactate concentrations can rise up to supraphysiological levels of 10–30 mM [43] induced by glycolytic cells that export lactate via MCT4. In agreement with our findings and those of other groups, the MCT1 F367Y mutant, which introduces a hydroxyl group to mimic Tyr332 of MCT4, decreased lactate affinity into this lactate concentration range. There are only a few functional studies on MCT3 [44]. It is found solely at the basolateral membrane of the retinal pigment epithelium where it enables transepithelial lactate transport together with the apical MCT1 [45]. MCT3 serves as a designated lactate exporter to the choroidal circulation.

In conclusion, the determination of the *k*_in_/*k*_out_‐ratio under metabolism‐free steady‐state assay conditions yielded novel insight into protein‐intrinsic MCT transport asymmetry. The data provide a structural and functional basis that contributes to the understanding of the various roles of the MCT isoforms in physiological settings.

## 4 Materials and Methods

### 4.1 Plasmids and Mutations

The expression constructs of human MCT1 with N‐terminal hemagglutinin‐ and C‐terminal His_10_‐tags in pDR196 (His3 selection marker) [14], and the l‐lactate‐biosensor iLACCO1 [46] in pRS413 (Ura3 selection marker) for co‐expression were described previously [47]. Site‐directed mutagenesis of the MCT1 coding sequence was done using primers listed in Table S1, and confirmed by sequencing.

### 4.2 Yeast Transformation and Culture

*S. cerevisiae* yeast lacking endogenous monocarboxylate transporters (W3031A‐1A jen1Δ ady2Δ; MATa leu2‐3112 trp1‐1 can1‐100 ura3‐1 ade2‐1 his3‐11,15 jen1::kanMX4 ady2::hphMX4), kindly provided by M. Casal [29], was transformed using the PEG/lithium acetate/single stranded carrier DNA protocol [48]. Transformed cells were selected at 29°C on SD agar supplemented with 2% (w/v) glucose, plus adenine, leucine, and tryptophan, but lacking either histidine or uracil or both for plasmid selection [47].

### 4.3 Hyperpolarized ^13^C‐NMR Spectroscopy

Dissolution dynamic nuclear polarization (dDNP) samples were prepared by mixing 14 M [1–^13^C]pyruvate (Sigma‐Aldrich, 677175, CAS: 99124‐30‐8) with 30 mM trityl radical (Polarize, AH111501) or 2.5 M [1–^13^C]lactate with 16 mM trityl radical, 280 mM glycerin (G9012, CAS: 56‐81‐5, Sigma‐Aldrich), and 2.4 mM gadolinium (gadobutrol Gd‐DO3A‐butrol, 1 mmol/mL; Gadovist, Bayer) in H_2_O (Carl Roth, ID 3478.1). The dissolution medium for pyruvate was prepared by mixing 1.51 g of Trizma pre‐set crystals (Sigma‐Aldrich, T7943), 27 mg of EDTA (SERVA, 11280.02), 0.756 g of NaCl (Sigma‐Aldrich, S98881), and 0.81 g of NaOH (Carl Roth, 9356.1) in 250 mL of H_2_O. For lactate, 300 mg of Trizma were combined with 3.9 mg of EDTA in 50 mL of H_2_O. No extra purification of the chemicals was performed. Hyperpolarization was performed using a cryogen‐free dDNP system (SpinAligner, Polarize), operating at about 1.3 K and 6.7 T with a microwave frequency between 186.995 and 187.045 GHz at 20 mW as detailed previously [49]. During each dDNP experiment, the polarization buildup (20 μL pyruvate or 50 μL lactate sample) was observed using ^13^C NMR in the solid state (ca. 1° excitation every 2–5 min), yielding characteristic time constants of about 20 min for pyruvate and 50 min for lactate. The samples were rapidly dissolved by adding 3.8 mL of superheated dissolution medium (pH of 7.5–7.6), and 280 μL were added to ca. 200 μL of yeast suspension inside a 5 mm NMR tube inside a 9.4 T NMR spectrometer (200 mg/mL cells in 0.2 M KH_2_PO_4_ yielding a pH 5.7–5.9, and 56 mM pyruvate or 21 mM lactate final concentrations, at 37°C; Bruker WB 400, 9.4 T, AvanceNeo, Paravision 360, BBFO probe). The time of detection was ca. 15 to 23 s after dissolution. The data was apodized by a 3 Hz exponential function in the time domain for the kinetics. Phase correction and a third‐order polynomial baseline correction was applied for each metabolite peak after Fourier transform and before numerical integration (MNova, 14.2.2, Mestrelab Research S.L.). The metabolism kinetics were analyzed using the Broyden–Fletcher–Goldfarb–Shanno algorithm to minimize the system of ordinary differential equations for the substrate and up to four products described previously [50].

### 4.4 Membrane Protein Extraction and Western Blotting

50 mL of yeast culture were grown to at an OD_600_ of 1.0 ± 0.1, harvested at 4000 *g*, 5 min, 4°C, and washed with 10 mL ice‐cold TE buffer (25 mM TRIS–HCl, 5 mM EDTA, pH 7.5). Cell pellets were stored at—80°C. For disruption, pellets were resuspended in 0.5 mL TE buffer and vortexed with 0.5 mL acid‐washed glass beads (Ø 425–600 μm, Sigma Aldrich) in 15 cycles of 30 s each. Glass beads and cell debris were removed by centrifugation at 1000 *g*, 5 min, 4°C, and subsequently at 10 000 *g*, 5 min, 4°C. The microsomal fraction was collected by ultracentrifugation at 100 000 *g*, 45 min, 4°C. The membrane pellets were resuspended in 100 μL of 100 mM Na_2_HPO_4_, pH 8.0, with 50 mM NaCl. 30 μg of total protein (Bradford assay, Bio‐Rad) were separated by SDS‐PAGE (12.5% acrylamide, peqGold III protein marker, VWR), and blotted on PVDF membrane (Hybond P 0.45, GE Healthcare). Detection was carried out by using a monoclonal mouse anti‐hemagglutinine antibody (Roche Diagnostics), a horseradish‐peroxidase‐conjugated secondary goat‐anti‐mouse antibody (Jackson ImmunoResearch) and freshly mixed ECL reagent (6.7 mM *p*‐coumaric acid, 1.3 mM luminol, 90 mM Tris–HCl, pH 8.6, 0.03% H_2_O_2_) in a ChemoStar Touch Imager (INTAS Science Imaging Instruments).

### 4.5 Transmembrane Transport Assay Using ^14^C‐l‐Lactate

Transport of radiolabeled l‐lactate was measured as described previously [51]. In brief, yeast cells were grown to an OD_600_ of 1.0 ± 0.1, harvested at 4000 *g*, 5 min, 4°C, washed with cold water, resuspended in 50 mM HEPES/Tris (pH 6.8) to an OD_600_ of 50 ± 5, and kept on ice. For the transport assay, 80 μL of cell suspension per sample were prewarmed to 19°C. Uptake was initiated by adding 20 μL of ^14^C‐spiked l‐lactate, propionate or pyruvate (Hartmann Analytics) solution (1 mM final concentration, 0.04 μCi/sample). For export measurements, the cell samples were incubated with ^14^C‐spiked lactate solution for 2–16 min until an intracellular lactate level of about 0.2 nmol mg^−1^ was reached. For Michaelis–Menten kinetics, l‐lactate solutions were added to yield final concentrations in the range of 0.5–20 mM with 0.04–0.12 μCi radiolabel per sample. At the indicated time points, transport was stopped by removal of the supernatant by vacuum filtration through GF/C glass microfiber filters, and washing with 7 mL of ice‐cold water. The filters were placed in 3 mL of scintillation cocktail (ROTISZINTeco plus, Carl Roth), and analyzed by liquid scintillation counting (Packard TriCarb 2900TR, Perkin Elmer). Cell‐adhering background radiolabel was determined from non‐expressing cells and subtracted.

### 4.6 Homology Modeling of MCT1

The experimental structure of open‐state MCT1 [26] solved at a resolution of 3.30 Å lacks coordinates for a 60 residue domain between transmembrane helices (TM) 6 and 7. We used ColabFold v.1.5.5 [52] with a custom template selection to generate structural models of wild‐type human MCT1 with this 60‐residue domain. As templates we used the structures of open‐state MCT1 bound to lactate (PDB# 6LZ0) and bound to an inhibitor (PDB ID: 6LYY) [26]. Following initial attempts to generate a full‐length MCT1 model, we removed from the sequence the disordered N‐ and C‐ termini and generated the model for the residue range G16 to A449. We inspected all generated models and chose the structural model with the smallest root mean square deviation (RMSD) value of 0.64 Å for the TM region of the model vs. experimental structure of open‐state MCT1. We added the Basigin protein and the lactate molecule from the experimental structure of the MCT1‐Basigin‐lactate complex (PDB ID: 6LZ0) [26]. To model the F367Y mutant we used CHARMM‐GUI [53]. All titratable sidechains were considered in their standard protonation sites and the lactate molecule was negatively charged.

### 4.7 Molecular Dynamics (MD) Simulations

We used the Positioning of Proteins in Membranes server [54] to orient the structure along membrane normal and CHARMM‐GUI Membrane Builder [53, 55, 56] to embed the Basigin‐MCT1‐lactate complex in a hydrated 1‐palmitoyl‐2‐oleoyl‐sn‐glycero‐3‐phosphocholine (POPC). The system was neutralized using a concentration of 0.15M of potassium chloride salt (KCl). We performed MD simulations using GROMACS 2023.2 [56, 57] with the CHARMM36m force field [58, 59, 60, 61] for protein, lipids, and ions, and the TIP3 water model [62]. The equilibration and geometry optimization were done using standard CHARMM‐GUI protocol. The equilibration was performed in NPT ensemble (constant number of particles N, constant pressure P, and constant temperature T), at a temperature of 303.15 K. We used a switching function between 10 and 12 Å for non‐bonded interactions, Particle Mesh Ewald (PME) for electrostatics, and an integration time step of 2 fs. We saved coordinates each 100 ps. We extended each simulation to 500 ns. Replica simulations for the WT and mutant MCT1 protein complexes were initiated from the same starting coordinates using different starting velocities (Figures S5 and S6).

### 4.8 Graph Computations of Lactate‐Protein‐Water Hydrogen Bond Networks

To compute hydrogen bond networks we used the graph‐based algorithm and graphical user interface Bridge/Bridge2 [33, 34]. Hydrogen bond graphs we report consist of nodes (lactate and protein sidechains) and edges (direct hydrogen bonds and water‐mediated bridges between the nodes). We identified hydrogen bonds using geometric criteria of hydrogen‐bond distance ≤ 3.5 Å and angle ≤ 60°. From each simulation we used 2000 equally spaced coordinate snapshots from the last 200 ns and computed hydrogen bonds graphs for direct hydrogen bonds and water mediated bridges with up to 3 hydrogen‐bonded water molecules in the bridge. Thus, the average length of a water‐mediated bridge between two graph nodes (two protein residues) can take values between 0.0 (direct hydrogen‐bond) and 3.0 (three hydrogen‐bonded water molecules). We used Connected Component Analyses [63] in Bridge [33] to extract, from each hydrogen‐bond graph, the hydrogen‐bond cluster of the lactate molecule; this cluster consists of the graph nodes and edges that connect to the node of the lactate. The average occupancy of a hydrogen‐bond (direct or water mediated) indicates the percentage of coordinate sets in which the hydrogen‐bond criteria are met. For clarity, we show only hydrogen bond connections that are present during at least 50% of the trajectory segments used for data analyses.

### 4.9 Determination of Inhibitor Activity

Pelleted yeast cells co‐expressing MCT1 wildtype or MCT1 M151A/F367Y/S371G with iLACCO1 were resuspended to an OD_600_ of 5 ± 0.5 in 50 mM HEPES/Tris, pH 6.8 and kept on ice. 80 μL of cell suspensions were incubated for 15 min at room temperature with 1 μL of inhibitor solution in DMSO (range 0–8 mM for AZD3965, or 0–20 mM for AZD0095) in 96‐well plates. In a plate reader (Spark, Tecan), 20 μL of l‐lactate solution were added well‐by‐well to establish an inward gradient of 1 mM. The cells were shaken at 510 rpm for 3 s and the change in fluorescence signal intensity, (*F*—*F*_0_)/*F*_0_, was monitored over 30 s (*λ*_ex_ 484 ± 10 nm, *λ*_em_ 515 ± 10 nm). Inhibitor activity was determined as IC_50_ after normalization of the fluorescence intensities obtained with uninhibited (100%) and fully blocked transporter (0%) [47].

### 4.10 Statistical Analysis

Curve fittings and graph visualizations were done using SigmaPlot (Systat Software). Lactate transport, Michaelis–Menten kinetics, and IC_50_ values were determined from three biological replicates, each in technical duplicates. Data represent mean ± SEM.

## Author Contributions

**Ioana‐Daniela Dumitru:** conceptualization, investigation, writing – original draft, methodology, validation, visualization, formal analysis, software. **Maike Menzel:** investigation, conceptualization, methodology, validation, visualization, writing – original draft, formal analysis, writing – review and editing. **Jan‐Bernd Hövener:** conceptualization, funding acquisition, validation, supervision, methodology, writing – original draft, data curation. **Josh Peters:** conceptualization, investigation, writing – original draft, methodology, validation, formal analysis, visualization. **Ana‐Nicoleta Bondar:** conceptualization, funding acquisition, writing – original draft, validation, visualization, methodology, software, supervision, formal analysis, resources, data curation. **Andrey N. Pravdivtsev:** conceptualization, investigation, validation, visualization, supervision, writing – original draft. **Eric Beitz:** conceptualization, funding acquisition, writing – original draft, methodology, validation, writing – review and editing, formal analysis, project administration, supervision, resources, data curation.

## Funding

This study was supported by the European Union's Horizon 2020/2024 research and innovation programmes under the Marie Skłodowska‐Curie grants PROTON no. 860592 and WATER no. 101227436 (to A.‐N.B and E.B.). I‐DD is supported by a doctoral scholarship from the Doctoral School of the Faculty of Physics of the University of Bucharest. The Authors gratefully acknowledge computing time on the supercomputer JURECA at the Forschungszentrum Jülich under grant no. DYNAMICNETWORKS. A.N.P., J.P., J.B.H. acknowledge funding from the German Federal Ministry of Education and Research (BMBF, 03WIR6208A hyperquant), DFG (555951950, 527469039, 469366436, HO‐4602/2‐2, HO‐4602/3, EXC2167, FOR5042, TRR287). MOIN CC was founded with a grant from the European Regional Development Fund (ERDF), the Zukunftsprogramm Wirtschaft of Schleswig‐Holstein (Project no. 122‐09‐053), the Medical Faculty, and Kiel University.

## Ethics Statement

The authors have nothing to report.

## Conflicts of Interest

The authors declare no conflicts of interest.

## Supporting information


**Figure S1:** Sequence alignment of lactate transporting hMCT1‐4.
**Figure S2:** Western blot showing expression of human MCT1 wildtype and mutants.
**Figure S3:** Extracted kinetics of hyperpolarized [1–^13^C]pyruvate and [1–^13^C]lactate.
**Figure S4:** Michaelis–Menten kinetics of MCT1 wildtype and mutants.
**Figure S5:** Lactate hydrogen bond clusters for MCT1 WT & F367Y; average length.
**Figure S6:** Lactate hydrogen bond clusters for MCT1 WT & F367Y; average occupancy.
**Figure S7:** Structure of a putative MCT1 methionine‐aromatic interaction.
**Table S1:** Mutation primers for MCT1 M151A, F367Y, and S371G variants.

## Data Availability

All data that support the findings of this study are available in the Supporting Information of this article.
