# Supplementary material for: Phenylalanine Versus Tyrosine (Pos. 367/332 in MCT1/MCT4) in the Substrate Binding Site Defines Affinity and Preferred Directionality of Human Monocarboxylate Transporters 1–4
Source: Acta Physiol (Oxf). 2026 Jun 14;242(7):e70267. doi: 10.1111/apha.70267 (PMC13265630; doi:10.1111/apha.70267)
Supplement: Supplementary file 1 — Figure S1: Sequence alignment of lactate transporting hMCT1‐4. Figure S2: Western blot showing expression of human MCT1 wildtype and mutants. Figure S3: Extracted kinetics of hyperpolarized [1–13C]pyruvate and [1–13C]lactate. Figure S4: Michaelis–Menten kinetics of MCT1 wildtype and mutants. Figure S5: Lactate hydrogen bond clusters for MCT1 WT & F367Y; average length. Figure S6: Lactate hydrogen bond clusters for MCT1 WT & F367Y; average occupancy. Figure S7: Structure of a putative MCT1 methionine‐aromatic interaction. Table S1: Mutation primers for MCT1 M151A, F367Y, and S371G variants. [file APHA-242-e70267-s001.pdf]

# Phenylalanine vs. tyrosine (pos. 367/332 in MCT1/MCT4) in the substrate binding site defines affinity and preferred directionality of human monocarboxylate transporters 1–4

Maike Menzel, Ioana-Daniela Dumitru, Josh Peters, Jan-Bernd Hövener, Andrey N. Pravdivtsev, Ana-Nicoleta Bondar, Eric Beitz

**Figure S1** Sequence alignment of lactate transporting hMCT1-4

**Figure S2** Western blot showing expression of human MCT1 wildtype and mutants

**Figure S3** Extracted kinetics of hyperpolarized [1-$^{13}$C]pyruvate and [1-$^{13}$C]lactate

**Figure S4** Michaelis-Menten kinetics of MCT1 wildtype and mutants

**Figure S5** Lactate hydrogen bond clusters for MCT1 WT & F367Y; average length

**Figure S6** Lactate hydrogen bond clusters for MCT1 WT & F367Y; average occupancy

**Figure S7** Structure of a putative MCT1 methionine-aromatic interaction

**Table S1** Mutation primers for MCT1 M151A, F367Y, and S371G variants

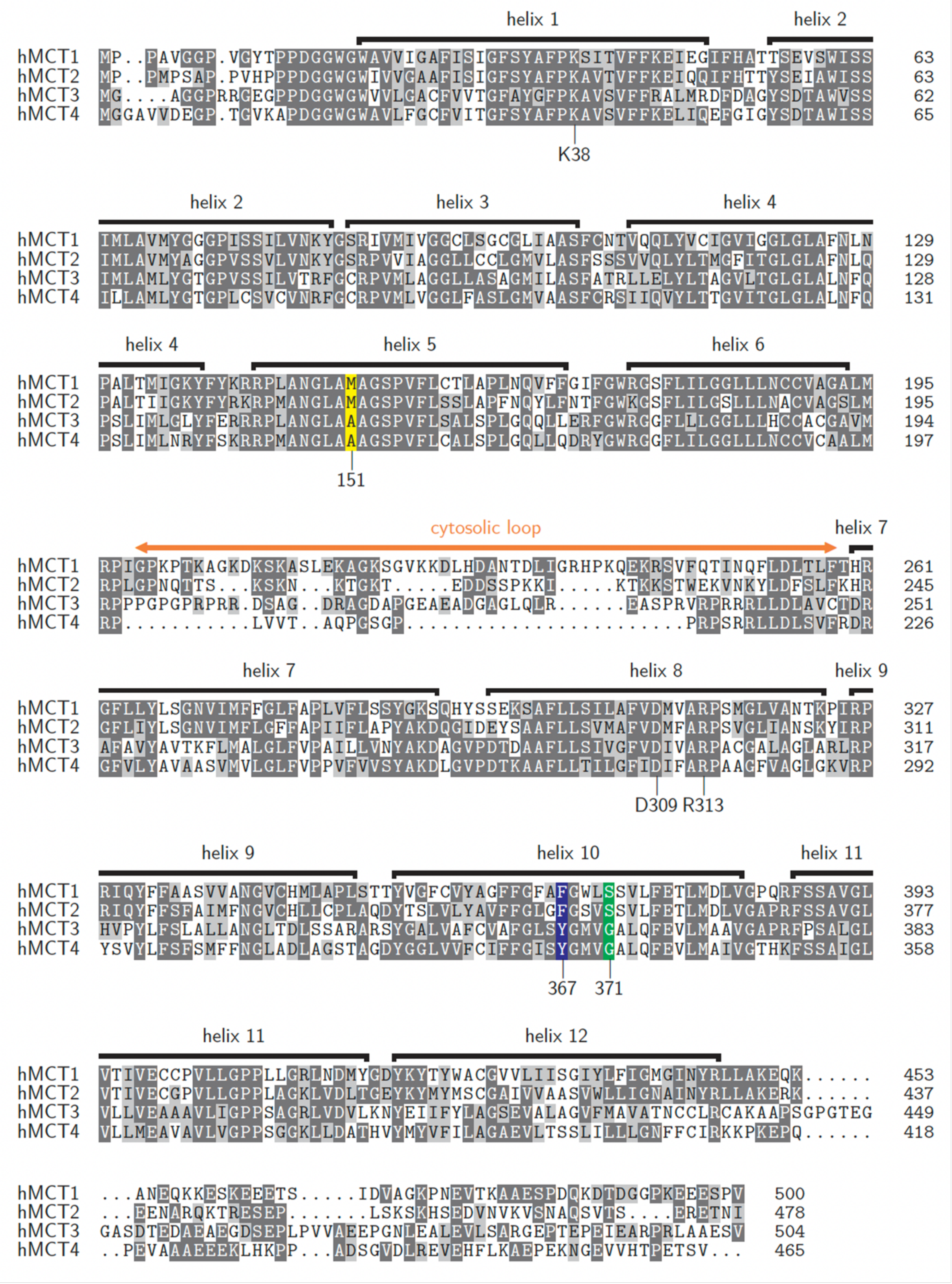

**Figure S1.** Sequence alignment of lactate transporting hMCT1-4. The amino acid positions differing in the transport pathways between MCT1/2 and MCT3/4, i.e. Met151, Phe367 and Ser371 (MCT1 numbering), are labeled and were subject of mutational analysis in this study.

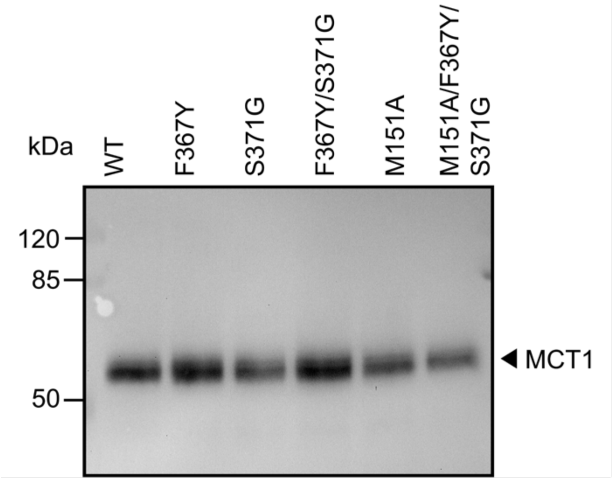


**Figure S2.** Western blot showing expression of human MCT1 wildtype (WT) and mutants in *S. cerevisiae* cells. For blotting the microsomal fraction was isolated, and 30 µg of total protein were loaded per lane. MCT1 protein was detected using an antibody directed against N-terminally fused hemagglutinin tag.

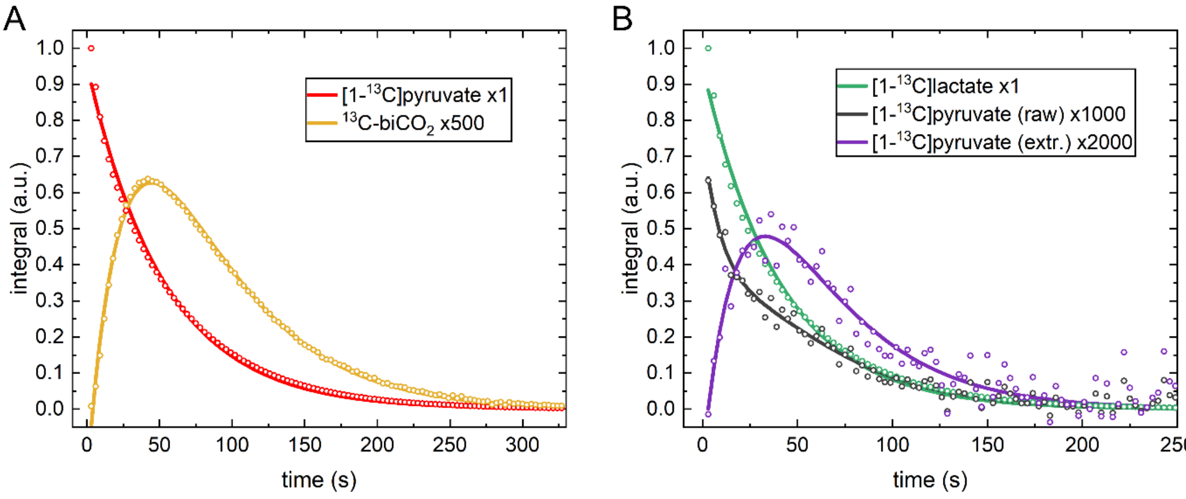


**Figure S3.** Extracted kinetics of hyperpolarized [1-$^{13}$C]pyruvate (A) and [1-$^{13}$C]lactate (B). (A) For analysis, the integrals of $CO_2$ and bicarbonate have been combined, yielding a rate constant of 3.02 ± 0.03·$10^{-4}$/s, which has been extracted with an initial pyruvate concentration of 56.4 mM. This leads to a conversion of 1.023 ± 0.009 mM pyruvate within one minute per 1 billion cells. (B) Due to contamination during the synthesis of hyperpolarized lactate, traces of [1-$^{13}$C]pyruvate are co-polarized (pool). However, a kinetic model was used to extract lactate to pyruvate kinetics from the underlying tri-exponential behavior (decaying co-polarized pyruvate pool, conversion of lactate to pyruvate, and decaying of converted pyruvate). The obtained $T_1$ values were 43.0 ± 1.1 s for the pyruvate pool and 23.3 ± 2.1 s for the created pyruvate, with the latter likely being intracellular and thus faster relaxing. A rate constant of 1.39 ± 0.16·$10^{-5}$/s has been extracted with an initial lactate concentration of 20.83 mM. This results in a conversion of 0.017 ± 0.002 mM lactate within one minute per 1 billion cells, which is 59 ± 8 times lower compared to the pyruvate conversion.

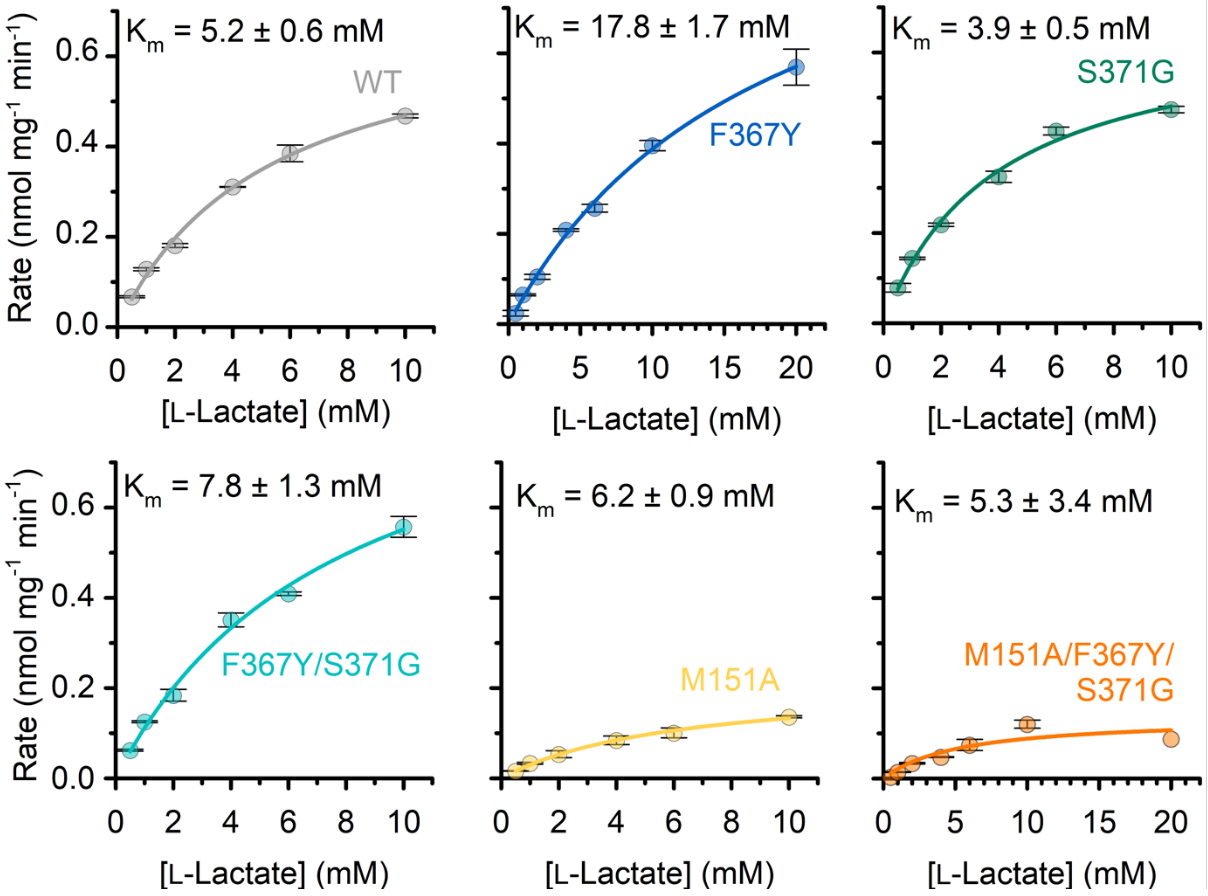


**Figure S4.** Michaelis-Menten kinetics of MCT1 wildtype and mutants. Rates of lactate uptake at increasing substrate concentrations were monitored at $pH_e$ 6.8. Error bars denote S.E.M

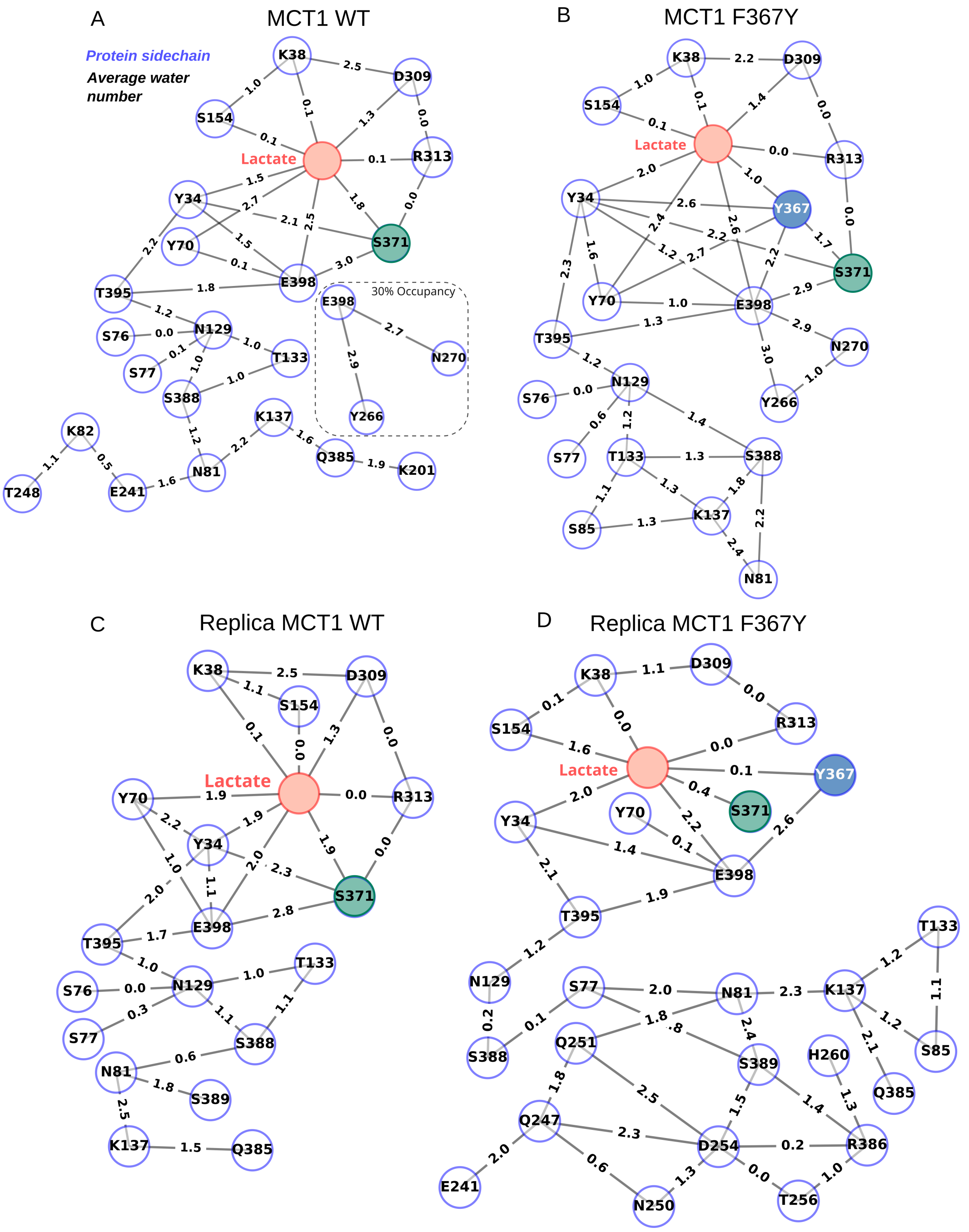


**Figure S5.** Lactate hydrogen bond clusters for MCT1 WT and F367Y, with average lengths of water bridges indicated along the graph edges. (A, C) MCT1 WT main simulation (panel A) and replica (panel C). (B, D) MCT1 F367Y mutant main simulation (panel B) and replica (panel D). Note that the lactate has very similar hydrogen bond connections in both of the WT simulations –mostly direct connections to K38, R313 and S154, short water bridges to D309 (1.3 waters in both simulations), and longer water bridge to S371 (1.8-1.9 waters) and E398 (~2-2.5 waters). Likewise, in both of the mutant simulations Y367 connects to the lactate via a direct or short (one water) bridge. The minimum hydrogen bond occupancy is 50%.

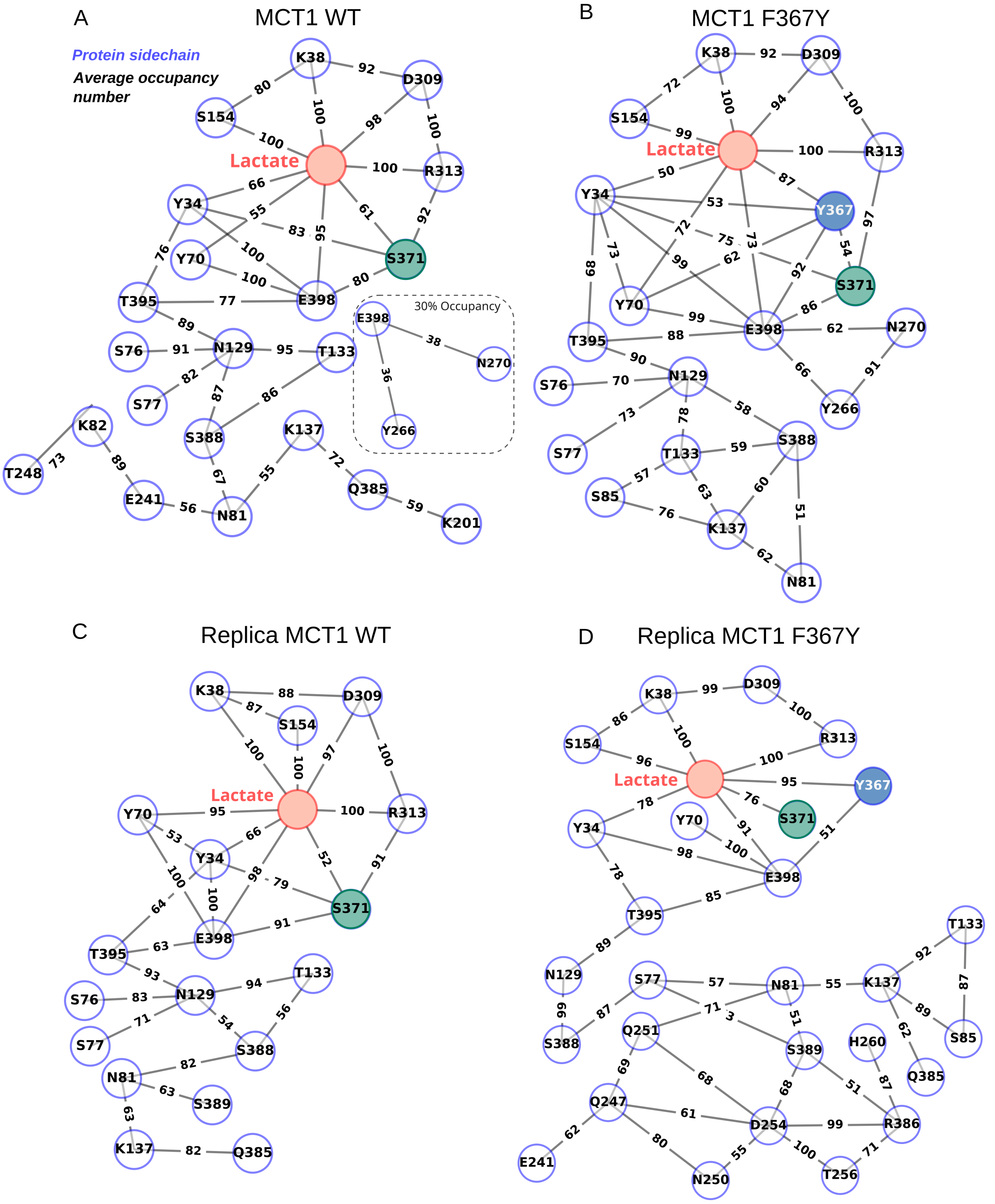


**Figure S6.** Lactate hydrogen bond clusters for MCT1 WT and F367Y, with average hydrogen bond occupancies indicated along the graph edges. (A, C) MCT1 WT main simulation (panel A) and replica (panel C). (B, D) MCT1 F367Y mutant main simulation (panel B) and replica (panel D). Note that both WT simulations indicate persistent (high occupancies) hydrogen bonds and water bridges for the lactate molecule. Likewise, both F367Y mutant simulations indicate persistent connections between Y367 and the lactate.

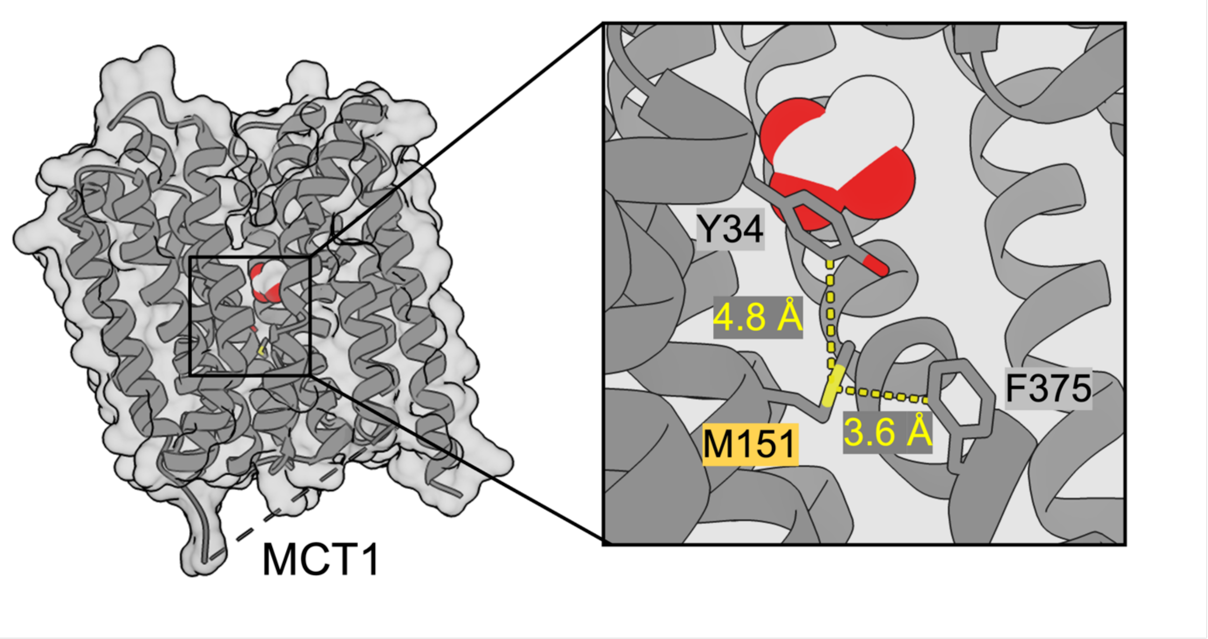


**Figure S7.** Structure of lactate-bound MCT1 (PDB# 6LZ0) showing the putative conformation-stabilizing methionine-aromatic interaction. Distances between Met151 and Tyr34 or Phe375 are indicated.

**Table S1**. Mutation primers for MCT1 M151A, F367Y, and S371G variants. Non-complementary nucleotides are underlined.

| | |
|---|---|
| M151A, sense | 5' AAC GGA CTG GCC GCG GCA GGC AGC CCT GT 3' |
| M151A, antisense | 5' GGC CAG TCC GTT GGC CAA TGG TCG CCT CTT 3' |
| F367Y, sense | 5' TTC TTT GGA TTT GCC TAC GGG TGG CTC AGC TCC3 ' |
| F367Y, antisense | 5' GGC AAA TCC AAA GAA TCC CGC ATA GAC ACA 3' |
| S371G, sense | 5' GCC TTC GGG TGG CTC GGC TCC GTA TTG TTT GAA 3' |
| S371G, antisense | 5' GAG CCA CCC GAA GGC AAA TCC AAA GAA TCC 3' |
| F367Y/S371G, sense | 5' TTC TTT GGA TTT GCC TAC GGG TGG CTC GGC TCC 3' |
